# Supplementary material for: Heat adaptation of phage T7 under an extended genetic code
Source: Virus Evol. 2021 Dec 1;7(2):veab100. doi: 10.1093/ve/veab100 (PMC8923235; doi:10.1093/ve/veab100)
Supplement: veab100_Supp [file veab100_supp.zip › Sequencing_Analysis.zip!/Sequencing_Analysis/S3/evidence/JC_SIDE_1_48.html]

BRESEQ :: Evidence


| New junction evidence | | | | | | | | | | | |
| --- | --- | --- | --- | --- | --- | --- | --- | --- | --- | --- | --- |
|  | | seq id | position | reads (cov) | reads (cov) | score | skew | freq | annotation | gene | product |
| \* | ? | NC\_001604.1 | = 8335 | 1786 (1.040) | 7 (0.000) | 6/584 | 44.4 | 0.39% | intergenic (–/–) | *–/–* | –/– |
|  | ? | NC\_001604.1 | 8939 = | 1866 (1.110) |  |  |  |  | intergenic (–/–) | *–/–* | –/– |
| Rejected: Coverage evenness skew score above cutoff. | | | | | | | | | | | |
| Rejected: Frequency below/above cutoff threshold. | | | | | | | | | | | |

Only 100 of 1794 total aligned reads displayed.  

`AGCGTCCACATCATTATCACCCGTGGTGACTTTGAGAAAGCAATAGACAAGAAACGTCCGGTTCTTAGTGTGGCAGTGACCCGCTTCCCGTTCGTCCGTCTGTTACTCAAACGAATCAAGGAGGTGTTCTGATGGGACTGTTAGATGGTGAAGCCTGGGAAAAAGAAAACCCGCCAGTACAAGCAACTGGGTGTATAGCTTGCTTAGAGAAAGATGACCGTTATCCACACACCTGTAACAAAGGAGCTAACGATATGACCGAACGTGAACAAGAGATGATCATTAAGTTGATAGACAATAATGAAGGTCGCCCAGATGATTTGAATGGCTGCGGTATTCTCTGCTCCAA‑TGTCCCTTGCCACCTCTGCCCCGCAAATAACGATCAAAAGATAACCTTAGGTGAAATCCGAGCGATGGACCCACGTAAACCACATCTGAATAAACCTGAGGTAACTCCTACAGATGACCAGCCTTCCGCTGAGACAATCGAAGGTGTCACTAAGCCTTCCCACTACATGCTGTTTGACGACATTGAGGCTATCGAAGTGATTGCTCGTTCAATGACCGTTGAGCAGTTCAAGGGATACTGCTTCGGTAACAT  >  NC_001604.1/8035‑8635`  

                                                                                                                                                                                                                                                                                                            `|`                                                                                                                                                                                                                                                                                                               
  `cGTCCACATCATTATCACCCGTGGTGACTTTGAGAAAGCAATAGACAAGAAACGTCCGGTTCTTAGTGTGGCAGTGACCCGCTTCCCGTTCGTCCGTCTGTTACTCAAACGAATCAAGGAGGTGTTCTGATGGGACTGTTAGATGGTGAAGCCTGGGAAAAAGAAAACCCGCCAGTACAAGCAACTGGGTGTATAGCTTGCTTAGAGAAAGATGACCGTTATCCACACACCTGTAACAAAGGAGCTAACGATATGACCGAACGTGAACAAGAGATGATCATTAAGTTGATAGACaataata                                                                                                                                                                                                                                                                                                             >  1:322829/1‑300 (MQ=255)`  
   `tgtgtataagagacagcccGTGGTGACTTTGAAAAAGCAATAGACACGAAATGTCCGGTTCTTAGTGTGGCAGGGACCCGCTTCCCGTTCGTCCGTCTGTAACTCAAACGAATCAAGGAGGTGTTCTGATGGGACTGTTAGATGGTGAAGCCTGGGAAAAAGAAAACCCGCCAGTACAAGCAACTGGGTGTATAGCTTGCTTAGAGAAAGATGACCGTTATCCACACACCTGTAACAAAGGAGCTAACGATATGACCGAACGTGAACAAGAGATGATCATTAAGTTGATAGACAATAATGa                                                                                                                                                                                                                                                                                                            <  1:346026/285‑1 (MQ=255)`  
   `gacagcatcatTATCACCCGTGGTGACTTTGAGAAAGCAATAGACAAGAAACGTCCGGTTCTTAGTGTGGCAGTGACCCGATTCCCGTTCGTCCGTCTGTTACTCAAACGAATCAAGGAGGTGTTCTGATGGGACTGTTAGATGGTGAAGCCTGGGAAAAAGAAAACCCGCCAGTACAAGCAACTGGGTGTATAGCTTGCTTAGAGAAAGATGACCGTTATCCACACACCTGTAACAAAGGAGCTAACGATATGACCGAACGTGAACAAGAGATGATCATTAAGTTGATAGACAATAATGa                                                                                                                                                                                                                                                                                                            <  1:255238/296‑1 (MQ=255)`  
    `tCCACATCATTATCACCCGTGGTGACTTTGAGAAAGCAATAGACAAGAAACGTCCGGTTCTTAGTGTGGCAGTGACCCGCTTCCCGTTCGTCCGTCTGTTACTCAAACGAATCAAGGAGGTGTTCTGATGGGACTGTTAGATGGTGAAGCCTGGGAAAAAGAAAACCCGCCAGTACAAGCAACTGGGTGTATAGCTTGCTTAGAGAAAGATGACCGTTATCCACACACCTGTAACAAAGGAGCTAACGATATGACCGAACGTGAACAAGAGATGATCATTAAGTTGTTAGACAATAATTaa                                                                                                                                                                                                                                                                                                           >  1:268736/1‑301 (MQ=255)`  
    `tCCACATCATTATCACCCGTGGTGACTTTGAGAAAGCAATAGACAAGAAACGTCCGGTTCTTAGTGTGGCAGTGACCCGCTTCCCGTTCGTCCGTCTGTTACTCAAACGAATCAAGGAGGTGTTCTGATGGGACTGTTAGATGGTGAAGACTGGGAAAAAGAAAACCCGCCAGTACAAGCAACTGGGTGTATAGCTTGCTTAGAGAAAGATGACCGTTATCCACACACCTGTAACAAAGGAGCTAACGATATGACCGAACGTGAACAAGAGATGATCATTAAGTTGATAGACAATAATGaa                                                                                                                                                                                                                                                                                                           >  1:314984/1‑301 (MQ=255)`  
     `aagacagatgaTCACCCGTGGTGACTTTGAGAAAGCAATAGACAAGAAACGTCCGGTTCTTAGTGTGGCAGTGACCCGCTTCCCGTTCGTCCGTCTGTTACTCAAACGAATCAAGGAGGTGTTCTGATGGGACTGTTAGATGGTGAGGCCGGGGAAAAAGAAAACCCGCCAGTACAAGCAACTGGGTGTATAGCTTGCTTAGAGAAGGATGACCGTTATCCACACACCTGTAACAAAGGAGCTAACGATATGACCGAACATGAACAAGAGATGATCATTAAGTTGATAGACAATAATGAAg                                                                                                                                                                                                                                                                                                          <  1:327396/291‑1 (MQ=255)`  
        `catcatTATCACCCGTGGTGACTTTGAGAAAGCAATAGACAAGAAACGTCCGGTTCTTAGTGTGGCAGTGACCCGCTTCCCGTTCGTCCGTCTGTTACTCAAACGAATCAAGGAGGTGTTCTGATGGGACTGTTAGATTGTGAAGCCTGGGAAAAAGAAAACCCGCCAGTACAAGCAACTGGGTGTATAGCTTGCTTAGAGAAAGATGACCGTTATCCACACACCTGTAACAAAGGAGCTAACGATATGACCGAACGTGAACAAGAGATGATCATTAAGTTGATAGTCAATAATGAAGGtt                                                                                                                                                                                                                                                                                                       >  1:155420/1‑300 (MQ=255)`  
        `catcatTATCACCCGTGGTGACTTTGAGAAAGCAATAGACAAGAAACGTCCGGTTCTTAGTGTGGCAGTGACCCGCTTCCCGTTCGTCCGTCTGTTACTCAAACGAATCAAGGAGGTGTTCTGATGGGACTGTTAGATGGTGAAGCCTGGGAAAAAGAAAACCCGCCAGTACAAGCAACTGGGTGTATAGCTTGCTTAGAGAAAGATGACCGTTATCCACACACCTGTAACAAAGGAGCTAACGATATGACCGAACGTGAACAAGAGATGATCATTAAGTTGATAGACAATAATGactgtc                                                                                                                                                                                                                                                                                                       >  1:287872/1‑296 (MQ=255)`  
        `catcatTATCACCCGTGGTGACTTTGAGAAAGCAATAGACAAGAAACGTCCGGTTCTTAGTGTGGCAGTGACCCGCTTCCCGTTCGTCCGTCTGTTACTCAAACGAATCAAGGAGGTGTTCTGATGGGACTGTTAGATGGTGAAGCCTGGGAAAAAGAAAACCCGCCAGTACAAGCAACTGGGTGTATAGCTTGCTTAGAGAAAGATGACCGTTATCCACACACCTGTAACAAAGGAGCTAACGATATGACCGAACGTGAACAAGAGATGATCATTAAGTTGATAGACAATAATGAAGGTc                                                                                                                                                                                                                                                                                                       >  1:48403/1‑301 (MQ=255)`  
                          `ggACTTTGAGAAAGAAATAGACAAGAAACGTCCGGTTCTTAGTGTGGCAGTGACCCGCTTCCCGTTCGTCCGTCTGTTACTCAAACGAATCAAGGAGGTGTTCTGATGAGACTGTTAGATGGTGAAGCCTGGGAAAAAGAAAACCCGCCAGTACAAGCAACTGGGTGTATAGCTTGCTTAGAGAAAGATGACCGTTATCCACACACCTGTAACAAAGGAGCTAACGATATGACCGAACGTGAACAAGAGATGATCATTAAGTTGATAGACAATAATGAAGGTCGCCCAGATGATTTGAATg                                                                                                                                                                                                                                                                                     <  1:58514/300‑1 (MQ=255)`  
                               `ttGAGAAAGCAATAGACAAGAAACGTCCGGTTCTTAGTGTGGCAGTGACCCGCTTCCCGTTCGTCCGTCTGTTACTCAAACGAATCAAGGAGGTGTTCTGATGGGACTGTTAGATGGTGAAGCCTGGGAAAAAGAAAACCCGCCAGTACAAGCAACTGGGTGTATAGCTTGCTTAGAGAAAGATGTCCGTTATCCACACACCTGTAACAAATGAGCTAACGATATGACCGAACGTGAACAAGAGATGATCATTAAGTTGATAGACAATAATGAAAGTCGCCCAGATGATTTGAATGGCTGc                                                                                                                                                                                                                                                                                >  1:293837/1‑301 (MQ=255)`  
                                `tGAGAAAGCAATAGACAAGAAACGTCCGGTTCTTAGTGTGGCAGTGACCCGCTTCCCGTTCGTCCGTCTGTTACTCAAACGAATCAAGGAGGTGTTCTGATGGGACTGTTAGATGGTGAAGCCTGGGAAAAAGAAAACCCGCCAGTACAAGCAACTGGGTGTATAGCTTGCTTAGAGAAAGATGACCGTTATCCACACACCTGTAACAAAGGAGCTAACGATATGACCGAACGTGAACAAGAGATGATCATTAAGTTGATAGACAATAATGAAGGTCGCCCAGCTGATTTGAATGGCTGCg                                                                                                                                                                                                                                                                               >  1:172215/1‑301 (MQ=255)`  
                                    `aaaGCAAAGGACAAGAAACGTCCGTTTCTTAGTGTGGCATGGACCCGCTTCCCGTTCGGCCGTCTGTTACGCAAAAGAATCAAGGAGGTGTTCTGATGGGACTGTTAGATGGTGAAGCCTGGGAAAAAGAAAACCCGCCAGTACAAGCAACTGGGTGTATAGCTTGCTTAGAGAAAGATGACCGTTATCCACACACCTGTAACAAAGGAGCTAACGATATGACCGAACGTGAACAAGAGATGATCATTAAGTTGATAGACAATAATGAAGGTCGCCCAGATGATTTGAATGGCTGCGGTAt                                                                                                                                                                                                                                                                           <  1:101884/301‑1 (MQ=255)`  
                                      `gcgcatgcaaaaGAACCCTCCAGTTCTTAGTGGGGCAGTGACCCGCTTCCCGTTCGTCCGTCTTTTACTCAAACGAAGCAAGGTGGTGTTCTGATGGGACTGTTAGATGGTGAAGCCTGGGAAAAAGAAAACCCGCCAGTACAAGCAACTGGGTGTATAGCTTGCTTAGAGAAAGATGACCGTTATCCACACACCTGTAACAAAGGAGCTAACGATATGACCGAACGTGAACAAGAGATGATCATTAAGTTGATAGACAATAATGAAGGTCGCCCAGATGATTTGAATGGCTGCGGTATtc                                                                                                                                                                                                                                                                         <  1:239650/291‑1 (MQ=255)`  
                                      `agaaaTAGACAAGAAACGGCCTGTTCTGAGGGTGTCAGTGACCCTCTTCCCGTACGACCGTATGTTAATCAAACGAATCAAGGATGTGTTCAGAAGAAACTGTTAGAAGGTGAAGCCTGGGAAAAGGAAAACCCGCCAATACAAGCAACTGGGTGTATAGCTTGCTTAGAGAATGATGACCATTATCCACACACCTGTAACAAAGGAGCTAACGATATGACCAAACGTGAACAAGAGATGATCATTAAGTTGATAGACAAGAATGAAGGTCGCCCAGATGATTTGAATTGCTGCGGTAtgc                                                                                                                                                                                                                                                                         <  1:274431/298‑3 (MQ=255)`  
                                       `gCAATAGACAAGAAACGTCCGGTTCTTAGTGTGGCAGTGACCCGCTTACCGTTCGTACGTCTGTTACTCAAACGAATCAAGGAGGTGTTCTGATGGGACTGTTAGATGGTGAAGCCTGGGAAAAAGAAAACCCGCCAGTACAAGCAACTGGGTGTATAGCTTGCTTAGAGAAAGATGACCGTTATCAACACACCTGTAACAAAGGAGCTAACGATATGACAGAACGTGAACAAGAGATGATAATTAAGTTGATAGACAATAATGAAGGTCGCCCAGATGATTTGAATGGCTGCGGTATtct                                                                                                                                                                                                                                                                        >  1:175819/1‑301 (MQ=255)`  
                                            `aGACAAGAAACGTCCGGTTCTTAGTGTGGCAGTGACCCGCTTCCCGTTCGTCCGTCTGTTACTCAAACGAATCAAGGAGGTGTTCTGATGGGACTGTTAGATGGTGAAGCCTGGG‑AAAAGAAAACCCGCCAGTACAAGCAACTGGGTGTATAGCTTGCTTAGAGAAAGATGACCGTTATCCACACACCTGTAACAAAGGAGCTAACGATATGACCGAACGTGAACAAGAGATGATCATTAAGTTGATAGACAATAATGAAGGTCGCCCAGATGATTTGAATGGCTGCGGTATTCTCTGCTc                                                                                                                                                                                                                                                                  >  1:136726/1‑301 (MQ=255)`  
                                             `gACAAGAAACGTCCGGTTCTTAGTGTGGCAGTGACCCGCTTCCCGTTCGTCCGTCTGTTACTCAAACGAATCAAGGAGGTGTTCTGATGGGACTGTTAGATGGTGAAGCCTGGGAAAAAGAAAACCCGCCAGTACAAGCAACTGGGTGTATAGCTTGCTTAGAGAAAGATGACCGTTATCCACACACCTGTAACAAAGGAGCTAACGATATGACCGAACGTGAACAAGAGATGATCATTAAGTTGATAGACAATAATGAAGGTCGCCCAGATGATTTGAATGGCTGCGGTATTCTCTGCTc                                                                                                                                                                                                                                                                  >  1:200551/1‑301 (MQ=255)`  
                                             `aaCAAGAAACTGCCGTTTTTGAGTGTGGCAGTGACACGCTTCCCGTTCGTCTGTCTGTTCCTCAAACGAATCAAGGAGGTGTTCTGAGGGGACTGTTAGATGTGTAAGCCTGGG‑AAAAGAAAACCCGCCAGTACAAGCAACTGGGTGTATAGCTTGCTTAGAGAAAGATGACCGTTATCCACACACCTGTAACAAAGGAGCTAACGATATGACCGAACGTGAACAAGAGATGATCATTAAGTTGATAGACAATAATGAAGGTCGCCCAGATGATTTGAATGGCTGCGGTATTCTCTGCTcc                                                                                                                                                                                                                                                                 <  1:166584/300‑1 (MQ=255)`  
                                                `aaGAACCGTCCGGTACTTAGTGTGGCAGTGACCCGCTTCCCGTTCGTCCGTCTGTAAATCAAACGAGTCAAGGAGGTGTTCTGATGGGACTGTTAGATGGTGAAGCCTGGGAAAAAGAAAACCCGCCAGTACAAGCAACTGTGTGTATAGCTTGCTTAGAGAAAGATGACCGTTATCCACACACCTGTAACAAAGGAGCTAACGATATGACCGAACGTGAACAAGAGATGATCATTAAGTTGATAGACAATAATGAAGGTCGCCCAGATGATTTGAATGGCTGCGGTATTCTCTGCTCCaa                                                                                                                                                                                                                                                               <  1:265447/301‑1 (MQ=255)`  
                                                 `aGAAACGTCCGGTTCTTAGTGTGGCAGTGACCCGCTTCCCGTTCGTCCGTCTGTTACTCAAACGAATCAAGGAGGTGTTCTGATGGGACTGTTAGATGGTGAAGACTGGGAAAAAGAAAACCCGCCAGTACAAGCAACTGGGTGTATAGCTTGCTTAGAGAAAGATGACCGTTATCCACACACCTGTAACAAAGGAGCTAACGATATGACCGAACGTGAACAAGAGATGATCATTAAGTTGATAGACAATAATGAAGGTCGCCCAGAGGAATTGAATGGCTGCGGGATTCTCTGcatgtct                                                                                                                                                                                                                                                              >  1:18801/1‑295 (MQ=255)`  
                                                            `gTTCTTAGTGTGGCAGTGACCCGCTTCCCGTTCGTCAGTCTGTTACTAAAACGAATCAAGGAGGTGTTCTGATGGGACTGTTAGATGGTGAAGCCTGGGAAAAAGAAAACCCGCCAGTACAAGCAACTGGGTGTATAGCTTGCTTAGAGAAAGATGACCGTTATACACACACCTGTAACAAAGGAGCTAACGATATGACCGAACGTGAACAAGAGATGATCATTAAGTTGATAGACAATAATGAAGGTAGACCCGATGATTTGAATGGCTGCGGTATTCTCTGCTCCAA‑TGTCACtgtctc                                                                                                                                                                                                                                                  >  1:92604/1‑296 (MQ=255)`  
                                                             `tacttaggtttgCAGTGACCCGCTTCCCGTTCGTCCGTCTGTTACTCAAACGAATCAAGGAGGTGTTCTGATGGGACTGTTAGATGGTGAAGCCTGGGAAAAAGAAAACCCTCCAGTACAAGCAACTGGGTGTATAGCTTGCTTAGAGAAAGATGACCGTTATCCACACACCTGTAACAAAGGAGCTAACGATATGACCGAACGTGAACAAGAGATGATCATTAAGTTGATAGACAATAATGAAGGTCGCCCAGATGATTTGAATGGCTGCGGTATTCTCTGCTCCAA‑TGTCCCTTGCCAc                                                                                                                                                                                                                                                 <  1:90103/290‑1 (MQ=255)`  
                                                              `tCTTAGTGTGGCAGTGACCCGCTTCCCGTTCGTCCGTCTGTTACTCAAACGAATCAAGGAGGTGTTCTGATGGGACTGTTAGATGGTGAAGCCTGGGAAAAAGAAAACCCGCCAGTACAAGCAACTGGGTGTATAGCTTGCTTAGAGAAAGATGACCGTTATCCACACACCTGTAACAAAGGAGCTAACGATATGACCGAACGTGAACAAGAGATGATCATTAAGTTGATAGACAATAATGAAGGTCGCCCAGATGATTTGAATGGCTGCGGTATTCTCTGCTCCAA‑TGACCCTTGCCAcc                                                                                                                                                                                                                                                >  1:43865/1‑301 (MQ=255)`  
                                                                `ttAGTGTGGCAGTGACCCGCTTCCCGTTCGTCCGTCTGTTACTCAAACGAATCAAGGAGGTGTTCTGATGGGACTGTTAGATGGTGAAGCATGGGAAAAAGAAAACCCGCCAGTACAAGCAACTGGGTGTATAGCTTGCTTAGAGAAAGATGACCGTTATCAATACACCTGTAACAAAGGAGCTAACGATATGACCGAACGTGAACAAGAGATGATCATTAAGTTGATAGACAATAATGAAGGTCGCCCAGATGATTTGAATGGCTGCGGTATTCTCTGCTCCAA‑TGTCCCTTGCCACctc                                                                                                                                                                                                                                              <  1:148482/301‑1 (MQ=255)`  
                                                                `ttAGTGTGGCAGGGACCCGATTCCCGTTAGTCAGTCTGTTACTCAAACGAATCAAGGAGGTGTTCTGATGGGACTGTTAGATGGTGAAGCCTGGGAAAAAGAAAACCCGCCAGTACAAGCAACTGGGTGTATAGCTTGCTTAGAGAAAGATGACCGTTATCCACACACCTGTAACAAAGGAGCTAACGATATGACCGAACGTGAACAAGAGATGATCATTAAGTTGATAGACAATAATGAAGGTCGCCCAGATGATTTGAATGGCTGCGGTATTCTCTGCTCCAA‑TGTCCCTTGCCACctc                                                                                                                                                                                                                                              <  1:76801/301‑1 (MQ=255)`  
                                                                `aaggtgtGGCATTGACCCGCTTCACGTTCTTCCGTCTGTTACTCAAACGAATCAAGGGGGTATGCTGATGGGACTGTAAGATGGTGAAACCTGGGAAAAAGAAAGCCCGCCAGTACAAGCAACTGGGTGTATAGCTTGCTTAGAGAAAGATGACCGTTATCCACACACCTGTAACAAAGGAGCTAACGATATGACCGAACGTGAACAAGAGATGATCATTAAGTTGATAGACAATAATGAAGGTCGCCCAGATGATTTGAATGGCTGCGGTATTCTCTGCTCCAA‑TGTCCCTTGCCACctc                                                                                                                                                                                                                                              <  1:30793/298‑1 (MQ=255)`  
                                                                        `gCAGTGACCCGCTTCCCGTTCGTCCGTCTGTTACTCAAACGAATCAAGGAGGTGTTCTGATGGGACTGTTAGATGGTGAAGCCTGGGAAAAAGAAAACCCGCCAGTACAAGCAACTGGGTGTATAGCTTGCTTAGAGAAAGATGACCGTTATCCACACATGTAAAACATAAGAGCACACGAGAAGCCAACGCGTACAAAAGAGATGATTAATAAGTATATAGAAAATAGTAAAGGTCACAAATATGTTTTGAGTCACGGTGGTTTTCTGCGCCACAA‑TGTCCCtctaccactccgccccca                                                                                                                                                                                                                                      >  1:139848/1‑284 (MQ=255)`  
                                                                           `gTGACCCGCTTCCCGTTCGTCCGTCTGTTACTCAAACGAATCAAGGAGGTGTTCTGATGGGACTGTTAGATGGTGAAGCCTGGGAAAAAGAAAACCCGCCAGTACAAGCAACTGGGTGTATAGCTTGCTTAGAGAAAGATGACCGTTATCCACACACCTGTAACAAAGGAGCTAACGATATGACCGAACGTGAACAAGAGATGATCATTAAGTTGATAGACAATAATGAAGGTCGCCCAGATGATTTGAATGGCTGCGGTATTCTCTGCTCCAA‑TGTCCCTTGCCACCTCTGCCCCGCaaa                                                                                                                                                                                                                                   >  1:304767/1‑301 (MQ=255)`  
                                                                           `gTGACCCGCTTCCCGTTCGTCCGTCTGTTACTCAAACGAATCAAGGAGGTGTTCTGATGGGACTGTTAGATGGTGAAGCCTGGGAAAAAGAAAACCCGCCAGTACAAGCAACTGGGTGTATAGCTTGCTTAGAGAAAGATGACCGTTATCCACACACCTGTAACAAAGGAGCTAACGATATGACCGAACGTGAACAAGAGATGATCATTAAGTTGATAGACAATAATGAAGGTCGCCCAGATGATTTGAATGGCTGCGGTATTCTCTGCTCCAA‑TGTCCCTTGCCACCTCTGCCCCGCaaa                                                                                                                                                                                                                                   >  1:86508/1‑301 (MQ=255)`  
                                                                               `cccGCTTCCCGTTCGTCCGTCTGTTACTCAAACGAATCAAGGAGGTGTTCTGATGGGACTGTTAGATGGTGAAGCCTGGGAAAAAGAAAACCCGCCAGTACAAGCAACTGGGTGTATAGCTTGCTTAGAGAAAGATGACCGTTATCCACACACCTGTAACAAAGGAGCTAACGATATGACCGAACGTGAACAAGAGATGATCATTAAGTTGATAGACAATAATGAAGGTCGCCCAGATGATTTGAATGGCTGCGGTATTCTCTGCTCCAA‑TGTCCCTTGCCACCTCTGCCCCGCAAATAAc                                                                                                                                                                                                                               <  1:282892/301‑1 (MQ=255)`  
                                                                                 `cGCTTCCCGTTCGTCCGTCTGTTACTCAAACGAATCAAGGAGGTGTTCTGATGGGACTGTTAGATGGTGAAGCCTGGGAAAAAGAAAACCCGCCAGTACAAGCAACTGGGTGTATAGCTTGCTTAGAGAAAGATGACCGTTATCCACACACCTGTAACAAAGGAGCTAACGATATGACCGAACGTGAACAAGAGATGATCATTAAGTTGATAGACAATAATGAAGGTCGCCCAGATGATTTGAATGGCTGCGGTATTCTCTGCTCCAACTGTCTCttatacacatctccgagcccacgaga                                                                                                                                                                                                                              >  1:263245/1‑277 (MQ=255)`  
                                                                                            `cgtccgtcTGTTACTCAAACGAATCAAGGAGGTGTTCTGATGGGACTGTTAGATGGTGAAGCCTGGGAAAAAGAAAACCCGCCAGTACAAGCAACTGGGTGTATAGCTTGCTTAGAGAAAGATGACCGGTATCCACACACCTGTAACAAAGGAGCTAACGATATGACCGAACGTGAACAAGAGATGATCATTAAGTTGATAGACAATAATGAAGGTCGCCCAGATGATTTGAATGGCTGCGGGATTCTCTGCTCCAA‑TGTCACTTGCCACCTCCGCCCCGTAAATAACGGTAAAAAGATaa                                                                                                                                                                                                                  >  1:190984/1‑301 (MQ=255)`  
                                                                                               `ccgtcTGTTACTCAAACAAATCAAGGAGGTGTGCTGATGGGACGGTTAGATGGTGAAGGCTGGGAAAAAGAAAACCCGCCAGTACAAGCAACTGGGTGTATAGCTTGCTTAGAGAAAGATGACCGTTATCCACACACCTGTAACAAAGGAGCTAACGATATGACCGAACGTGAACAAGAGATGATCATTAAGTTGATAGACAATAATGAAGGTCGCCCAGATGATTTGAATGGCTGCGGTATTCTCTGCTCCAA‑TGTCCCTTGCCACCTCTGCCCCGCAAATAACGATCAAAAGATAACCt                                                                                                                                                                                                               <  1:62742/301‑1 (MQ=255)`  
                                                                                                `ggtcTGTTACTAAAACGAATCAAGGAGGTGTTCTGATGGGACTGTTAGATGGTGAAGCCTGGGAAAAAGAAAACCCGCCAGTACAAGCAACTGGGTGTATAGCTTGCTTAGAGAAAGATGACCGTTATCCACACACCTGTAACAAAGGAGCTAACGATATGACTGAACGTGAACAAGAGATGATCATTAAGTTGATAGACAATAATGAAGGTCGCCCAGATGATTTGAATGGCTGCGGTATTCTCTGCTCCAA‑TGTCCCTTGCCACCTCTGCCCCGCAAATAACGATCAAAAGATAACCtt                                                                                                                                                                                                              <  1:145821/300‑1 (MQ=255)`  
                                                                                                `cagcTGTTACTAAAACGAATCAAGGAGGTGTTCTGATGGGACTGTTAGATGGTGAAGCCTGGGAAAAAGAAAACCCGCCAGTACAAGCAACTGGGTGTATAGCTTGCTTAGAGAAAGATGACCGTTAGCCACACACCTGTAACAAAGGAGCTAACGATATGACCGAACGTGAACAAGAGATGATCATTAAGTTGATAGACAATAATGAAGGTCGCCCAGATGATTTGAATGGCTGCGGTTTTCTCTGCTCCAA‑TGTCCCTTGCCACCTCTGCCCCGCAAATAACGATCAAAAGATAACCtt                                                                                                                                                                                                              <  1:100024/298‑1 (MQ=255)`  
                                                                                                          `tCAAACGAATCAAGGAGGTGTGCTGATGGGACTGATGGATGGTGAAGCCTAGGAAAAAGAAAACCCGCCAGTACAAGCAACTGGGTGTATAGCTTGCTTAGAGAAAGATGACCGTTATCCACACACCTGTAACAAAGGAGCTAACGATATGACCGAACGTGAACAAGAGATGATCATTAAGTTGATAGACAATAATGAAGGTCGCCCAGATGATTTGAATGGCTGCGGTATTCTCTGCTCCAA‑TGTCCCTTGCCACCTCTGCCCCGCAAATAACGATCAAAAGATAACCTTAGGTGAAATc                                                                                                                                                                                                    <  1:236841/301‑1 (MQ=255)`  
                                                                                                             `aaCGAATCAAGGAGGTGTTCTGATGGGACTGTTAGATGGTGAAGCCTGGGAAAAAGAAAACCCGCCAGTACAAGCAACTGGGTGTATAGCTTGCTTAGAGAAAGATGACCGTTATCCACACACCTGTAACAAAGGAGCTAACGATATGACCGAACGTGAACAAGAGATGATCATTAAGTTGATAGACAATAATGAAGGTCGCCCAGATGATTTGAATGGCTGCGGTATTCTCTGCTCCAA‑TGTCCCTTGCCACCTCTGCCCCGCAAATAACGATCAAAAGATAACC‑TAGGTGAAATCCGAg                                                                                                                                                                                                >  1:82866/1‑301 (MQ=255)`  
                                                                                                                 `aaTCAAGGAGGTGTTCTGATGGGACTGTTAGATGGTGAAGCCTGGGAAAAAGAAAACCCGCCAGTACAAGCAACTGGGTGTATAGCTTGCTTAGAGAAAGATGACCGTTATCCACACACCTGTAACAAAGGAGCTAACGATATGACCGAACGTGAACAAGAGATGATCATTAAGTTGATAGACAATAATGAAGGTCGCCCAGATGATTTGAATGGCTGCGGTATTCTCTGCTCCAA‑TGTCCCTTGCCACCTCTGCCCCGCAAATAACGATCAAAAGATAACCTTAGGTGAAATCCGAGCGa                                                                                                                                                                                             >  1:1851/1‑301 (MQ=255)`  
                                                                                                                           `agacagtGATGGGACTGTTAGATGGTGAAGCCTGGGAAAAAGAAAACCCGCCAGTACAAGCAACTGGGTGTATAGCTTGCTTAGAGAAAGATGACCGTTATCCACACACCTGTAACAAAGGAGCTAACGATATGACCGAACGTGAACAAGAGATGATCATTAAGTTGATAGACAATAATGAAGGTCGCCCAGATGATTTGAATGGCTGCGGTATTCTCTGCTCCAA‑TGTCCCTTGCCACCTCTGCCCCGCAAATAACGATCAAAAGATAACCTTAGGTGAAATCCGAGCGATGGACCCACg                                                                                                                                                                                   <  1:213585/295‑1 (MQ=255)`  
                                                                                                                            `tgtTCTGATGGGACTGTTAGATGGTGAAGCCTGGGAAAAAGAAAACCCGCCAGTACAAGCAACTGGGTGTATAGCTTGCTTAGAGAAAGATGACCGTTATCCACACACCTGTAACAAAGGAGCTAACGATATGACCGAACGTGAACAAGAGATGATCATTAAGTTGATAGACAATAATGAAGGTCGCCCAGATGATTTGAATGGCTGCGGTATTCTCTGCTCCAA‑TGTCCCTTGCCACCTCTGCCCCGCAAATAACGATCAAAAGATAACCTTAGGTGAAATCCGAGCGATGGACCCACGt                                                                                                                                                                                  <  1:13157/301‑1 (MQ=255)`  
                                                                                                                             `gtTCTGATGGGACTGTTAGATGGTGAAGCCTGGGAAAAAGAAAACCCGCCAGTACAAGCAACTGGGTGTATAGCTTGCTTATAGAAAGATGACCGTTATCCACACACCTGTAACAAAGGAGCTAACGATATGACCGAACGTGAACAAGAGATGATCATTAAGTTGATAGACAATAATGAAGGTCGCCCAGATGATTTGAATGGCTGCGGTATTCTCTGCTCCAA‑TGTCCCTTGCCACCTCTGCCCCGCAAATAACGATCAAAAGATAACCTTAGGTGAAATCCGAGCGATGGACCCACGTa                                                                                                                                                                                 >  1:332928/1‑301 (MQ=255)`  
                                                                                                                             `gtTCTGATGGGACTGTTAGATGGTGAAGCCTGGGAAAAAGAAAACCCGCCAGTACAAGCAACTGGGTGTATAGCTTGCTTAGAGAAAGATGACCGTTATCCACACACCTGTAACAAAGGAGCTAACGATATGACCGACCGTGAACAAGAGATGATCATTAAGTTGATAGACAATAATGAAGGTCGCCCAGATGATTTGAATGGCTGCGGTATTCTCTGCTCCAA‑TGTCCCTTGCCACCTCTGCCCCGCAAATAACGATCAAAAGATAACCTTAGGTGAAATCCGAGCGATGGACCCACGTa                                                                                                                                                                                 >  1:142906/1‑301 (MQ=255)`  
                                                                                                                             `gtTCTGATGGGACTGTTAGATGGTGAAGCCTGGGAAAAAGAAAACCCGCCAGTACAAGCAACTGGGTGTATAGCTTGCTTAGAGAAAGATGACCGTTATCCACACACCTGTAACAAAGGAGCTAACGATATGACCGAACGTGAACAAGAGATGCTCATTAAGTTGATAGACAATAATGAAGGTCGCCCAGATGATTTGAATGGCTGCGGTATTCTCTGCTCCAA‑TGTCCCTTGCCACCTCTGCCCCGCACATAACGATCAAAAGATAACCTTAGGTCAAATCCGAACCATGGACCCACATa                                                                                                                                                                                 >  1:179436/1‑301 (MQ=255)`  
                                                                                                                             `gtTCTGATGGGACTGTTAGATGGTGAAGCCTGGGAAAAAGAAAACCCGCCAGTACAAGCAACTGGGTGTATAGCTTGCTTAGAGAAAGATGACCGTTATCCACACACCTGTAACAAAGGAGCTAACGATATGACCGAACGTGAACAAGAGATGATCATTAAGTTGATAGACAATAATGAAGGTCGCCCAGATGATTTGAATGGCTGCGGTATTCTCTGCTCCAA‑TGTCCCTTGCCACCTCTGCCCCGCAAATAACGATCAAAAGATAACCtgtctcttatacacatctccgagcccacgag                                                                                                                                                                                 >  1:320585/1‑271 (MQ=255)`  
                                                                                                                                 `tGATGGGCCTGTTAGGTGGTGAAGCCTGGGAAAAAGAAAACCCGCCAGTACAAGCAACTGGGTGTATAGCTTGCTTAGAGAAAGATGACCGTTATCCACACACCTGTAACAAAGGAGCTAACGATATGACCGAACGTGAACAAGAGATGATCATTAAGTTGATAGACAATAAAGAAGGTCGCCCAGATGATTTGAATGGCTGCGGTATTCTCTGCTCCAA‑TGTCCCTTGCCACCTCTGCCCCGCAAATAACGATCAAAAGATAACCTTAGGTGAAATCCGAGCGATGGACCCACGTAAAcc                                                                                                                                                                             <  1:198092/301‑1 (MQ=255)`  
                                                                                                                                 `ggaaggtaaggTTATATGGTGAAGCAGGGGAAAAAGAAAACCCGCCAGTACAAGCAACGGGGTGTATCGCTTGCGTAGAGAAAGATGACCGGTAGCCACACACCTGTAACAAAGGAGCTAACGAGATGACCGAACGTGAACAAGAGATGATCATTAAGTTGATAGACAATAATGAAGGTCGCCCAGATGATTTGAATGGCTGCGGTATTCTCTGCGCCAA‑TGTCCCTTGCCACCTCTGCCCCGCAAATAACGATCAAAAGATAACCTTAGGTGAAATCCGAGCGATGGACCCACGTAAAcc                                                                                                                                                                             <  1:339006/291‑1 (MQ=255)`  
                                                                                                                                 `aaagagacaggTTAGATGGTGAAGCCTGGGAAAAAGAAAACCCGCCAGTACAAGCAACTGGGTGTATAGCTTGCTTAGAGAAAGATGACCGTTATCCACACACCTGTAACAAAGGAGCTAACGATATGACCGAACGTGAACAAGAGATGATCATTAAGTTGATAGACAATAATGAAGGTCGCCCAGATGATTTGAATGGCTGCGGTATTCTCTGCTCCAA‑TGTCCCTTGCCACCTCTGCCCCACAAATAACGATCAAAAGATAACCTTAGGTGAAATCCGAGCGATGGACCCACGTAAAcc                                                                                                                                                                             <  1:298084/291‑1 (MQ=255)`  
                                                                                                                                      `ttgtataagagacagaaaGCCTGGGAAAAAGAAAACCCGCCAGTACAAGCAACTGGGTGTATAGCTTGCTTAGAGAAAGATGACCGTTATCCACACACCTGTAACAAAGGAGCTAACGATATGACCGAACGTGAACAAGAGATGATCATTAAGTTGATAGACAATAATGAAGGTCGCCCAGATGATTTGAATGGCTGCGGTATTCTCTGCTCCAA‑TGTCCCTTGCCACCTCTGCCCCGCAAATAACGATCAAAAGATAACCTTAGGTGAAATCCGAGCGATGGACCCACGTAAACCACATc                                                                                                                                                                        <  1:330456/285‑1 (MQ=255)`  
                                                                                                                                           `gTTAGATGGTGAAGCAGGGGAAAAAGAAAACCCGCCAGTACAAGCAACTGGGTGTATAGCTTGCTTAGAGAAAGATGACCGTTATCCACACACCTGTAACAAAGGAGCTAACGATATGACCGAACGTGAACAAGAGATGATCATTAAGTTGATAGACAATAATGAAGGTCGCCCAGATGATTTGAATGGCTGCGGTATTCTCTGCTCCAA‑TGTCCCTTGCCACCTCTGCCCCGCAAATAACGATCAAAAGATAACCTTAGGTGAAATCCGAGCGATGGACCCACGTAAACCACATCTGAAt                                                                                                                                                                   <  1:68984/301‑1 (MQ=255)`  
                                                                                                                                                `agggggAAGCCGGGGAAAAAGAAAACCCGCCAGTACAAGCAACTGGGTGTATAGCTTGCTTAGACAAAGATGACCGTTATCCACACACCTGTAACAAAGGAGCTAACGATATGACCGAACGTGAACAAGAGATGAGCATTAAGTTGATAGACAATAATGAAGGTCGCCCAGATGATTTGAATGGCTGCGGTATTCTCTGCTCCAA‑TGTCCCTTGCCACCTCTGCCCCGCAAATAACGATCAAAAGATAACCTTAGGTGAAATCCGAGCGATGGACCCACGTAAACCACATCTGAATAAAcc                                                                                                                                                              <  1:134109/296‑1 (MQ=255)`  
                                                                                                                                                `aTGGTGAAGCATGGGAAAAAAAAAACCCGCCAGTACAAGCAACTGGGTGTATAGCTTGCTTAGAGAAAGATGACCGTTATCCACACACCTGTAACAAAGGAGCTAACGATATGACCGAACGTGAACAAGAGATGATCATTAAGTTGATAGACAATAATGAAGGTCGCCCAGATGATTTGAATGGCTGCGGTATTCTCTGCTCCAA‑TGTCCCTTGCCACCTCTGCCCCGCAAATAACGATCAAAAGATAACCTTAGGTGAAATCCGAGCGATGGACCCACGTAAACCACATCTGAATAAAcc                                                                                                                                                              <  1:162734/301‑1 (MQ=255)`  
                                                                                                                                                  `ggTGAAGCCTGGGAAAAAAAAAACCCGCCAGTACAAGCAACTGGAGGTATAGCTTGCTTAGAGAAAGATGACCGTTATCCACACACCTGTAACAAAGGAGCTAACGATATGACCGAACGTGAACAAGAGATGATCATTAAGTTGATAGACAATAATGAAGGTCGCCCAGATGATTTGAATGGCTGCGGTATTCTCTGCTCCAA‑TGTCCCTTGCCACCTCTGCCCCGCAAATAACGATCAAAAGATAACCTTAGGTGAAATCCGAGCGATGGACCCACGTAAACCACATCTGAATAAACCTg                                                                                                                                                            <  1:336459/301‑1 (MQ=255)`  
                                                                                                                                                       `aGCCTGGGAAAAAGAAAACCCGCCAGTACAAGCAACTGGGTGTATAGCTTGCTTAGAGAAAGATGACCGTTATCCACACACCTGTAACAAAGGAGCTAACGATATGACCGAACGTGAACAAGAGATGATCATTAAGTTGATAGACAATAATGAAGGTCGCCCAGATGATTTGAATGGCTGCGGTATTCTCTGCTCCAA‑TGTCCCTTGCCACCTCTGCCCCGCAAATAACGATCAAAAGATAACCTTAGGTGAAATCCGAGCGATGGACCCACGTAAACCACATCTGAATAAACCTGAGGTa                                                                                                                                                       <  1:222999/301‑1 (MQ=255)`  
                                                                                                                                                                 `gtgggctcggagatgtgtataagagacaggTGTATAGCTTGCTTAGAGAAAGATGACCGTTATCCACACACCTGTAACAAAGGAGCTAACGATATGACCGAACGTGAACAAGAGATGATCATTAAGTTGATAGACAATAATGAAGGTCGCCCAGATGATTTGAATGGCTGCGGTATTCTCTGCTCCAA‑TGTCCCTTGCCACCTCTGCCCCGCAAATAACGATCAAAAGATAACCTTAGGTGAAATCCGAGCGATGGACCCACGTAAACCACATCTGAATAAACCTGAGGTAACTCCTACAg                                                                                                                                             <  1:195243/273‑1 (MQ=255)`  
                                                                                                                                                                 `aaaGCAAACCCGCTAGTACAAGCAACTGGGTGTATAGCTTGCTTAGAGAAAGATGATCTTTATCCACACACCTGTAACAAAGGAGCTAACGATATGACCGAACGTGAATAAGAGATGATTATTAAGTTGATAGCCAATAAGGAAGGTCGCCCAGATGATTTGAATGGCTGCGGGATTCTCTGCTCCAA‑TGTCCCTTGCCACCTCTGCCCCGCAAATAACGATCAAAAGATAACCTTAGGTGAAATCCGAGCGATGTACCCACGTAAACCACATCTGAATAAACCTGAGGTAACTCCTACAg                                                                                                                                             <  1:54849/301‑1 (MQ=255)`  
                                                                                                                                                                      `aaaCCCGCCAGTACAAGCAACTGGGTGTATAGCTTGCTTAGAGAAAGATGACCGTTATCCACACACCTGTAACAAAGGAGCTAACGATATGACCGAACGTGAACAAGAGATGATCATTAAGTTGATAGACAATAATGAAGGTCGCCCAGATGATTTGAATGGCTGCGGTATTCTCTGCTCCAA‑TGTCCCTTGCCACCTCTGCCCCGCAAATAACGATCAAAAGATAACCTTAGGTGAAATCCGAGCGATGGACCCACGTAAACCACATCTGAATAAACCTGAGGTAACTCCTACAGCTgtc                                                                                                                                        >  1:22583/1‑299 (MQ=255)`  
                                                                                                                                                                       `aaCCCGCCAGTACAAGCAACTGGGTGTATAGCTTGCTTAGAGAAAGATGACCGTTATCCACACACCTGTAACAAAGGAGCTAACGATATGACCGAACGTGAACAAGAGATGATCATTAAGTTGATAGACAATAATGAAGGTCGCCCAGATGATTTGAATGGCTGCGGTATTCTCTGCTCCAA‑TGTCCTTTGCCACCTCTGCCCCGCAAATAACGATCAAAAGATAACCTTAGGTGAAATCCGAGCGATGGACCCACGTAAACCACATCTGAATAAACCTGAGGTAACTCCTACAGATGAcc                                                                                                                                       <  1:66273/301‑1 (MQ=255)`  
                                                                                                                                                                           `cGCCAGTACAAGCAACTGGGTGTATAGCTTGCTTAGAGAAAGATGACCGTTATCCACACACCTGTAACAAAGGAGCTAACGATATGACCGAACGTGAACAAGAGATGATCATTAAGTTGATAGACAATAATGAAGGTCGCCCAGATGATTTGAATGGCTGCGGTATTCTCTGCTCCAA‑TGTCCCTTTCCACCTCTGCCCCGCAAATAACGATCAAAAGATAACCTTAGGTGAAATCCGAGCGATGGACCCACGTAAACCACATCTGAATCAACCTGAGGTAACTCCTACAGATGATCAGcc                                                                                                                                   >  1:342151/1‑301 (MQ=255)`  
                                                                                                                                                                                `gTACAAGCAACTGGGTGTATAGCTTGCTTAGAGAAAGATTACCGTTATCCACACACCTGTAACAAAGGAGCTAACGATATGACCGAACGTGAACAAGAGATGATCATTAAGTTGATAGACAATAATGAAGGTCGCCCAGATGATTTGAATGGCTGCGGTATTCTCTGCTCCAA‑TGTCCCTTGCCACCTCTGCCCCGCAAATAACGATCAAAAGATAACCTTAGGTGAAATCCGAGCGATGGACCCACGTAAACCACATCTGAATAAACCTGAGGTAACTCCTACAGATGACCAGCCTTCCg                                                                                                                              >  1:25936/1‑301 (MQ=255)`  
                                                                                                                                                                                            `gggTGTATAGCTTGCTTAGAGAAAGATGACCGTTATCCACACACCTGTAACAAAGGAGCTAACGATATGACCGAACGTGAACAAGAGATGATCATTAAGTTGATAGACAATAATGAAGGTCGCCCAGATGATTTGAATGGCTGCGGTATTCTCTGCTCCAA‑TGTCCCTTGCCACCTCTGCCCCGCAAATAACGATCAAAAGATAACCTTAGGTGAAATCCGAGCGATGGACCCACGTAAACCACATCTGAATAAACCTGAGGTAACTCCTACAGATGACCAGCCTTCCGCTGAGACAATCg                                                                                                                  >  1:205854/1‑301 (MQ=255)`  
                                                                                                                                                                                                    `aGCTTGCTTAGAGAAAGATGACCGTTATCCACACACCTGTAACAAAGGAGCTAACGATATGACCGAACGTGAACAAGAGATGATCATTAAGTTGATAGACAATAATGAAGGTCGCCCAGATGATTTGAATGGCTGCGGTATTCTCTGCTCCAA‑TGTCCCTTGCCACCTCTGCCCCGCAAATAACGATCAAAAGATAACCTTAGGTGAAATCCGAGCGATGGACCCACGTAAACCACATCTGAATAAACCTGAGGTAACTCCTACAGATGACCAGCCTTCCGCTGAGCCAATCGAAGGTGTc                                                                                                          >  1:106830/1‑301 (MQ=255)`  
                                                                                                                                                                                                    `aGCTTGCTTAGAGAAAGATGACCGTTATCCACACACCTGTAACAAAGGAGCTAACGATATGACCGAACGTGAACAAGAGATGATCATTAAGTTGATAGACAATAATGAAGGTCGCCCAGATGATTTGAATGGCTGCGGTATTCTCTGCTCCAA‑TGTCCCTTGCCACCTCTGCCCCGCAAATAACGATCAAAAGATAACCTTAGGTGAAATCCGAGCGATGGACCCACGTAAACCACATCTGAATAAACCTGAGGTAACTCCTACAGATGACCAGCCTTCCGCTGAGACAATCGAAGGTGTc                                                                                                          >  1:216632/1‑301 (MQ=255)`  
                                                                                                                                                                                                       `ttgcttAGAGAAAGATGACCGTTATCCACCAACCTGTAACAAAGGAGCTAACGATATGACCGAACGTGAACAAGAGATGATCATTAAGTTGATAGACAATAATGAAGGTCGCCCAGATGATTTGAATGGCTGCGGTATTCTCTGCTCCAA‑TGTCCCTTGCCACCTCTGCCCCGCAAATAACGATCAAAAGATAACCTTAGGTGAAATCCGAGCGATGGACCCACGTAAACCACATCTGAATAAACCTGAGGTAACTCCTACAGATGACCAGCCTTCCGCTGAGACAATCGAAGGTGTCACt                                                                                                       <  1:233140/301‑1 (MQ=255)`  
                                                                                                                                                                                                                   `aGATGACCGTTATCCACACACCTGTAACAAAGGAGCTAACGATATGACCGAACGTGAACAAGAGATGATCATTAAGTTGATAGACAATAATGAAGGTCGCCCAGATGATTTGAATGGCTGCGGTATTCTCTGCTCCAA‑TGTCCCTTGCCACCTCTGCCCCGCAAATAACGATCAAAAGATAACCTTAGGTGAAATCCGAGCGATGGACCCACGTAAACCACATCTGAATAAACCTGAGGTAACTCCTACAGATGACCAGCCTTCCGCTGAGACAATCGAAGGTGTCACTAAGCCTTCCCAc                                                                                           <  1:270867/301‑1 (MQ=255)`  
                                                                                                                                                                                                                    `gATGACCGTTATCCACACACCTGTAACAAAGGAGCTAACGATATGACCGAACGTGAACACGAGATGATCATTAAGTTGATAGACAATAATGACGGTCGCCCAGATGATTTGAATGGCTGCGGTATTCTCTGCTCCAA‑TGTCCCTTGCCACCTCTGCCCCGCAAATAACGATCAAAAGATAACCTTAGGTGAAATCCGAGCGATGGACCCACGTAAACCACATCTGAATAAACCTTCGTTAACTCCTACAGATTACCAGCCTTCCGCTGAGACAATCAAAGGTGTCACTCAGCCTTCCCACt                                                                                          >  1:31187/1‑301 (MQ=255)`  
                                                                                                                                                                                                                      `tGACCGTTATCCACACACCTGTAACAAAGGAGGTAACGATATGACCGAACGTGAACAAGAGATGATAATTAAGTTGATAGACAATAATGAAGGTCGCCCAGATGATTTGAATGGCTGCGGTATTCTCTGCTCCAA‑TGTCCCTTGCCACCTCTGCCCCGCAAATAACGATCAAAAGATAACCTTAGGTGAAATCCGAGCGATGGACCCACGTAAACCACATCTGAATAAACCTGAGGTAACTCCTACAGATGACCAGCCTTCCGCTGAGACAATCGAAGGTGTCACTAAGCCTTCCCACTAc                                                                                        <  1:94677/301‑1 (MQ=255)`  
                                                                                                                                                                                                                        `aCCGTTATCCACACACCTGTAACAAAGGAGCTAACGATATGACCGAACGTGAACAAGAGATGATCATTAAGTTGATAGACAATAATGAAGGTCGCCCAGATGATTTGAATGGCTGCGGTATTCTCTGCTCCAA‑TGTCCCTTGCCACCTCTGCCCCGCAAATAACGATCAAAAGATAACCTTAGGTGAAATCCGAGCGATTGACCCACGTAAACCACATCTGAATAAACCTGAGGTAACTCCTACAGATGACCAGCCTTCCGCTGAGACAATCGAAGGTGTCACTAAGCCTTCCCACTACAt                                                                                      >  1:71372/1‑301 (MQ=255)`  
                                                                                                                                                                                                                         `ccGTTATCCACACACCTGTAACAAAGGAGCTAACGATATGACCGAACGTGAACAAGAGATGATCATTAAGTTGATAGACAATAATGAAGGTCGCCCAGATGATTTGAATGGCTGCGGTATTCTCTGCTCCAA‑TGTCCCTTGCCACCTCTGCCCCGCAAATAACGATCAAAAGATAACCTTAGGTGAAATCCGAGCGATGGACCCACGTAAACCACATCTGAATAAACCTGAGGTAACTCCTACAGATGACCAGCCTTCCGCTGAGACAATCGAAGGTGTCACTAAGCCTTCCCACTACATg                                                                                     >  1:119311/1‑301 (MQ=255)`  
                                                                                                                                                                                                                          `cGTTATCCACACACCTGTAACAAAGGAGCTAACGATATGACCGATCGTGAACAAGAGATGATCATTAAGTTGATAGACAATAATGAAGGTCGCCCAGATGAATTGAATGGCTGCGGTATTCTCTGCTCCAA‑TGTCCCTTGCCACCTCTGCCCCGCAAATAACGATCAAAAGATAACCTTAGGTGAAATCCGAGCGATGGACCCACGTAAACCACATCTGAATAAACCTGAGGTAACTCCTACAGATGACCAGCCTTCCGCTGAGACAATCGAAGGTGTCACTAAGCCTTCCCACTACATGc                                                                                    <  1:40947/301‑1 (MQ=255)`  
                                                                                                                                                                                                                           `gTTATCCACACACCTGTAACAAAGGAGCTAACGATATGACCGAACGTGAACAAGAGATGATCATTAAGTTGATAGACAATAATGAAGGTCGCCCAGATGATTTGAATGGCTGCGGTATTCTCTGCTCCAA‑TGTCCCTTGCCACCTCTGCCCCGCAAATAACGATCAAAAGATAACCTTAGGTGAAATCCGAGCGATGGACCCACGTAAACCACATCTGAATAAACCTGAGGTAACTCCTACAGATGACCAGCCTTCCGCTGAGACAATCGAAGGTGTCACTAAGCCTTCCCACTACATGCt                                                                                   <  1:158603/301‑1 (MQ=255)`  
                                                                                                                                                                                                                                `ccACACACCTGTAACAAAGGAGCTAACGATATGACCGAACGTGAACAAGAGATGATCATTAAGTTGATAGACAATAATGAAGGTCGCCCAGATGATGTGAATGGCTGCGGTATTCTCTGCTCCAA‑TGTCCCTTGCCACCTCTGCCCCGCAAATAACGATCAAAAGAGAACCATAGGTGACATCCGAGCGATGGAACAACGTAAACCAAATCTGAATAAACCTGAGGTAACTCCTACAGATGACCAGCCATCCGCTGAGACAATCGAAGGGGTCAATAAGCCTTCCCACTACATTCAGtttt                                                                              >  1:219286/1‑300 (MQ=255)`  
                                                                                                                                                                                                                                  `acacacCTGTAACAAAGGAGCTAACGATATGACCGAACGTGAACAAGAGATGATCATTAAGTTGATAGACAATAATGAAGGTCGCCCAGATGATTTGTATGGCTGCGGTATTCTCTGCTCCAA‑TGTCCCTTGCCACCTCTGCCCCGCAAATAACGATCAAAAGATAACCTTAGGTGAAATCCGAGCGATGGACCCACGTAAACCACATCTGAATAAACCTGAGGTAACTCCTACAGATGACCAGCCTTCCGCTGAGACAATCGAAGGTGTCACTAAGCCTTCCCACTACATGCTGTTTgac                                                                            <  1:74631/301‑1 (MQ=255)`  
                                                                                                                                                                                                                                       `ccTGTAACAAAGGAGCTAACGATATGACCGAACGTGAACAAGAGATGATCCTTACGTTGATAGACAATAATGAAGGTCGCCCAGATGATTTGAATGGCTTCGGTCTTCTCTGCTCCAC‑TGTCCCTTGCCACCTCTGCCCCGCCCCTACCGATCAAAAGATGACCTTAGGTGAAATCCGAGCGATGGACCCACGTAAACCACATCTGAATAAACCTGAGGTAACTCCTACAGATGACCAGCCTTCCGCTGGGACAATCGAAGGTGTCACTAAGCCTTCCCCCTCACTTCTGTTTGACGACAt                                                                       >  1:27956/1‑301 (MQ=255)`  
                                                                                                                                                                                                                                       `ccTGTAACAAAGGAGCTAACGATATGACCGAACGTGAACAAGAGATGATCATTAAGTTGATAGACAATAATGAAGGTCGCCCAGATGATTTGAATGGCTGCGGTATTCTCTGCTCCAA‑TGTCCCTTGCCACCTCTGCCCCGCAAATAACGATCAAAAGATAACCTTAGGTGAAATCCGAGCGATGGACCCACGTAAACCACATCTGAATAAACCTGAGGTAACTCCTACAGATGACCAGCCTTCCGCTGAGACAATCGAAGGTGTCACTAAGCCTTCCCACTACATGCTGTTTGACGACAt                                                                       >  1:209766/1‑301 (MQ=255)`  
                                                                                                                                                                                                                                          `gTAACAAAGGAGCTAACGATATGACCGAACGTGAACAAGAGATGATCATTAAGTTGATAGACAATAATGAAGGTCGCCCAGATGATTTGAATGGCTGCGGTATTCTCTGCTCCAA‑TGTCCCTTGCCACCTCTGCCCCGCAAATAACGATCAAAAGATAACCTTAGGTGAAATCCGAGCGATGGACCCACGTAAACCACATCTGAATAAACCTGAGGTAACTCCTACAGATGACCAGCCTTCCGCTGAGACAATCGAAGGTGTCACTAAGCCTTCCCACTACATGCTGTTTGACGACCTTGa                                                                    >  1:252764/1‑301 (MQ=255)`  
                                                                                                                                                                                                                                             `aacagggAGCAAACGATATGTCCGAACGTGGACAAGAGATGATGATTAAGTTGAAAGACAAGAATGAAGGTCGCCCAGAGGATTTGAAGGGGTGCAGAATTCGATGCTAAAA‑TGTCCCTTGCCAACTGTGACGCGCAAATAACGATCAAAAGATAAACTTAGGTGAAATACGAGCGATGGAACCACTTAAACCACATCTGAATAAACCGGAGGTAACTACTACATATGACCAGCCTTCCGCTGAGACAATCGAAGGTGTCACTAAGCCTTCCCACTACATGCTGTTTGACGACATTGAGGc                                                                 <  1:317407/296‑1 (MQ=255)`  
                                                                                                                                                                                                                                              `cAAAGGAGCTAACGATATGACCGAACGTGAACAAGAGATGATCATTAAGTTGATAGACAATAATGAAGGTCGCCCAGATGATTTGAATGGCTGCGGTATTCTCTGCTCCAA‑TGTCCCTTGCCACCTCTGCCCCGCAAATAACGATCAAAAGATAACCTTAGGTGAAATCCGAGCGATGGACCCACGTAAACCACATCTGAATAAACCTGAGGTAACTCCTACAGATGACCAGCCTTCAGCTGAGACAATCGAAGGTGTCACTAAGCCTTCCCACTACATGCTGTTTgacgctgtctcttat                                                                >  1:151675/1‑290 (MQ=255)`  
                                                                                                                                                                                                                                                       `aagagacaggACCGAACGTGAACAAGAGATGATCATTAAGTTGATAGACAATAATGAAGGTCGCCCAGATGATTTGAATGGCTGCGGTATTCTCTGCTCCAA‑TGTCCCTTGCCACCTCTGCCCCGCAAATAACGATCAAAAGATAACCTTAGGTGAAATCCGAGCGATGGACCCACGTAAACCACATCTGAATAAACCTGAGGTAACTCCTACAGATGACCAGCCTTCCGCTGAGACAATCGAAGGTGTCACTAAGCCTTCCCACTACATGCTGTTTGACGACATTGAGGCTATCGAAGTg                                                       <  1:276935/292‑1 (MQ=255)`  
                                                                                                                                                                                                                                                            `atatGACCGAACGTGAACAAGAGATGATCATTAAGTTGATAGACAATAATGAAGGTCGCCCAGATGATTTGAATGGCTGCGGTATTCTCTGCTCCAA‑TGTCCCTTGCCACCTCTGCCCCGCAAATAACGATCAAAAGATAACCTTAGGTGAAATCCGAGCGATGGACCCACGTAAACCACATCTGAATAAACCTGAGGTAACTCCTACAGATGACCAGCCTTCCGCTGAGACAATCGAAGGTGTCACTAAGCCTTCCCACTACATGCTGTTTGACGACATTGAGGCTATCGAAGTGATTGc                                                  <  1:256896/301‑1 (MQ=255)`  
                                                                                                                                                                                                                                                             `gacagtcattattgtctatcaactgatCATTAAGTTGATAGACAATAATGAAGGTCGCCCAGATGATTTGAATGGCTGCGGTATTCTCTGCTCCAA‑TGTCCCTTGCCACCTCTGCCCCGCAAATAACGATCAAAAGATAACCTTAGGTGAAATCCGAGCGATGGACCCACGTAAACCACATCTGAATAAACCTGAGGTAACTCCTACAGATGACCAGCCTTCCGCTGAGACAATCGAAGGTGTCACTAAGCCTTCCCACTACATGCTGTTTGACGACATTGAGGATATCGAAGTGATTGCt                                                 <  1:109791/278‑1 (MQ=255)`  
                                                                                                                                                                                                                                                                      `aaagagaCAGGAGATGATCATTAAGTTGATAGACAATAATGAAGGTCGCCCAGATGATTTGAATGGCTGCGGTATTCTCTGCTCCAA‑TGTCCCTTGCCACCTCTGCCCCGCAAATAACGATCAAAAGATAACCTTAGGTGAAATCCGAGCGATGGACCCACGTAAACCACATCTGAATAAACCTGAGGTAACTCCGACAGATGACCAGCCTTCCGCTGAGACAATCGAAGCTGTCACTAAGCCTTCCCACTACATGCTGTTTGACGACATTGAGGCTATCGAAGTGAATGCTCGTTCAATg                                        <  1:249223/295‑1 (MQ=255)`  
                                                                                                                                                                                                                                                                        `gTGAACAAGAGATGATCATTAAGTTGATAGACAATAATGAAGGTCGCCCAGATGATTTGAATGGCTGCGGTATTCTCTGCTCCAA‑TGTCCCTTGCCACCTCTGCCCCGCAAATAACGATCAAAAGATAACCTTAGGTGAAATCCGAGCGATGGACCGACGTAAACCACACCTGAATAAACCTGAGGTAACTCCTACAGATGACCAGCCTTCCGCTGAGACAATCGAAGGTGTCACTAAGCCTTCGCACTACATGCTGTTTGACTACATTGAGGCTATCGACGTGGATGCTCGTTCAATGAc                                      >  1:12346/1‑301 (MQ=255)`  
                                                                                                                                                                                                                                                                                `gagaTGATCATTAAGTTGATAGACAATAATGAAGGTCGCCCAGATGATTTGAATGGCTGCGGTATTCTCTGCTCCAA‑TGTCCCTTGCCACCTCTGCCCCGCAAATAACGATCAAAAGATAACCTTAGGTGAAATCCGAGCGATGGACCCACGTAAACCACATCTGAATAAACCTGAGGTAACTCCTACAGATGACCAGCCTTCCGCTGAGACAATCGAAGGTGTCACTAAGCCTTCCCACTACATGCTGTTTGACGACATTGAGGCTATCGAAGTGATTGCTCGTTCAATGACCGTTGAGc                              <  1:51232/301‑1 (MQ=255)`  
                                                                                                                                                                                                                                                                                `gagaTGATCATTAAGTTGATAGACAATAATGAAGGTCGCCCAGATGATTTGAATGGCTGCGGTATTCTCTGCTCCAA‑TGTCCCTTGCCACCTCTGCCCCGCAAATAACGATCAAAAGATAACCTTAGGTGAAATCCGAGCGATGGACACACGTAAACCACATCTGAATAAACCTGAGGTAACTCCTACAGATGACCAGCCTTCCGCTGAGACAATCGAAGGTGTCACTAAGCCTTCCCACTACATGCTGTTTGACGACATTGAGGCTATCGAAGTGATTGCTCGTTCAATGACCGTTGAGc                              >  1:301973/1‑301 (MQ=255)`  
                                                                                                                                                                                                                                                                                      `atCATTAAGTTGATAGACAATAATGAAGGTCGCCCAGATGATTTGAATGGCTGCGGTATTCTCTGCTCCAA‑TGTCCCTTGCCACCTCTGCCCCGCAAATAACGATCAAAAGATAACCTTAGGTGAAATCCGAGCGATGGACCCACGTAAACCACATCTGAATAAACCTGAGGTAACTCCTCCAGATGACCAGCCTTCCGCTGAGACAATCGAAGGTGTCACTAAGCCTTCCCACTACATGCTGTTTGACGACATTGAGGCTATCGATGTGATTGCTCGTTCAATGACCGTTGCGCAGTTCa                        >  1:35534/1‑301 (MQ=255)`  
                                                                                                                                                                                                                                                                                       `tCATTAAGTTGATAGACAATAATGAAGGTCGCCCAGATGATTTGAATGGCTGCGGTATTCTCTGCTCCAA‑TGTCCCTTGCCACCTCTGCCCCGCAAATAACGATCAAAAGATAACCTTAGGTGAAATCCGAGCGATGGACCCACGTAAACCACATCTGAATAAACCTGAGGTAACTCCTACAGATGACCAGCCTTCCGCTGAGACAATCGAAGGTGTCACTAAGCCTTCCCACTACATGCTGTTTGACGACATTGAGGCTATCGAAGTGATTGCTCGTTCAATGACCGTTGAGCAGTTCaa                       >  1:245235/1‑301 (MQ=255)`  
                                                                                                                                                                                                                                                                                       `tCATTAAGTTGATAGACAATAATGAAGGTCGCCCAGATGATTTGAATGGCTGCGGTATTCTCTGCTCCAA‑TGTCCCTTGCCACCTCTGCCCCGCAAATAACGATCAAAAGATAACCTTAGGTGAAATCCGAGCGATGGACCCACGTAAACCACATCTGAATAAACCTGAGGTAACTCCTACAGATGACCAGCCTTCCGCTGAGACAATCGAAGGTGTCACTAAGCCTTCCCACTACATGCTGTTTGACGACATTGAGGCTATAGAAGTGATTGCTCGTTAAATGACCGTTGAGCAGTTCaa                       >  1:116301/1‑301 (MQ=255)`  
                                                                                                                                                                                                                                                                                          `tgaaagtGATATACAATACAGAAGGTCTCTCGGATGATTTGAATGGCTGCGGTATTCTCTGCTCCAA‑TGTCCCTTGCCACCTCTGCCCCGCAAATAACGATCAAATGATAACCTTAGGTGAAATCTGAGCGATGGACCCACGTAAACCACATCTGAATATACCTGAGGTAACTCCTACAGATGACCAGCCTGCCGCTGAGCCAATCGAAGGTGTCACTAGGCCTTCCCACTACATGCTGTTTGACGACAGTGAGGCTATCGAAGTGATTGCTTGTTCAATGACCGTTGAGCAGTTCAAggg                    <  1:29116/295‑1 (MQ=255)`  
                                                                                                                                                                                                                                                                                          `acaggTTGATAGACAATAATGAAGGTCGCCAAGATGATTTGAATGGCTGCGGTATTCTCTGCTACAA‑TGTCCCTTGCCACCTCAGCCCCGCAAATAACGGTCAAAAGATAACCTTAGGTGAAATCCGAGCGATGGACCCACGTAAACCACATCTGAATAAACCTGAGGTAACTCCTACAGATGACCAGCCTTCCGCTGAGACAATCGAAGGTGTCACTAAGCCTTCCCACTACATGCTGTTTGACGACATTGAGGCTATCGAAGTGATTGCTCGTTCAATGACCGTTGAGCAGTTCAAggg                    <  1:285281/297‑1 (MQ=255)`  
                                                                                                                                                                                                                                                                                              `gTTGATAGACAATAATGAAGGTCGCCCAGATGATTTGAATGGCTGCGGTATTCTCTGCTCCAA‑TGTCCCTTGCCACCTCTGCCCCGCAAATAACGATCAAAAGATAACCTTAGGTGAAATCCGAGCGATGGACCCACGTAAACCACATCTGAATAAACCTGAGGTAACTCCTCCAGATGACCAGCCTTCCGCTGAGACAATCGAAGGTGTCACTAAGCCTTCCCACTACATTCTGTTTGACGACATTGAGGCTATCTAAGTGATTGCTCGTTCAATGACCGTTTAGCAGTTCAAGGGAtcc                >  1:104509/1‑299 (MQ=255)`  
                                                                                                                                                                                                                                                                                              `gTTGATAGACAATAATGAAGGTCGCCCAGATGATTTGAATGGCTGCGGTATTCTCTGCTCCAA‑TGTCCCTTGCCACCTCTGCCCCGCAAATAACGATCAAAAGATAACCTTAGGTGAAATCCGAGCGATGGACCCACGTAAACCACATCTGAATAAACCTGAGGTAACTCCTACAGATGACCAGCCTTCCGCTGAGACAATCGAAGGTGTCACTAAGCCTTCCCACTACATGCTGTTTGACGACATTGAGGCTATCGAAGTGATTGCTCGTTCAATGACCGTTGAGCAGTTCAAgggctgt                >  1:113574/1‑297 (MQ=255)`  
                                                                                                                                                                                                                                                                                                       `cAATAATGAAGGTCGCCCAGATGATTTGAATGGCTGCGGTATTCTCTGCTCCAA‑TGTCCCTTGCCACCTCTGCCCCGCAAATAACGATCAAAAGATAACCTTAGGTGAAATCCGAGCGATGGACCCACGTAAACCACATCTGAATAAACCTGAGGTAACTCCTACAGATGACCAGCCTTCCGCTGAGACAATCGAAGGTGTCACTAAGCCTTCCCACTACATGCTGTTTGACGACATTGAGGCTATCGAAGTTATTGCTCGTTCAATGACCGTTGAGCAGTTCAAGGGATACTGCTTCGGt       >  1:228632/1‑301 (MQ=255)`  
                                                                                                                                                                                                                                                                                                       `cAATAATGAAGGTCGCCCAGATGATTTGAATGGCTGCGGTATTCTCTGCTCCAA‑TGTCCCTTGCCACCTCTGCCCCGCAAATAACGATCAAAAGATAACCTTAGGTGAAATCCGAGCGATGGACCCACGTAAACCACATCTGAATAAACCTGAGGTAACTCCTACAGATGACCAGCCTTCCGCTGAGACAATCGAAGGTGTCACTAAGCCTTCCCACTACATGCTGTTTGACGACATTGAGGCTATCGAAGTGATTGCTCGTTCAATGACCGTTGAGCAGTTCAAGGGATACTGCTTCGGt       >  1:79501/1‑301 (MQ=255)`  
                                                                                                                                                                                                                                                                                                       `cAATAATGAAGGTCGCCCAGATGATTTGAATGGCTGCGGTATTCTCTGCTCCAA‑TGTCCCTTGCCACCTCTGCCCCGCAAATAACGATCAAAAGATAACCTTAGGTGAAATCCGAGCGATGGACCCACGTAAACCACATCTGAATAAACCTGAGGTAACTCCTACAGATGACCAGCCTTCCGCTGAGACAATCGAAGGTGTCACTAAGCCTTCCCACTACATGCTGTTTGACGACATTGAGGCTATCGAAGTGATTGCTCGTTCAATGACCGTTGAGCAGTTCAAGGGATACTGCTTCGGt       >  1:17030/1‑301 (MQ=255)`  
                                                                                                                                                                                                                                                                                                        `aataatGAAGGTCGCCCAGATGATTTGAATGGCTGCGGTATTCTCTGCTCCAA‑TGTCCCTTGCCACCTCTGCCCCGCAAATAACGATCAAAAGATAACCTTAGGTGAAATCCGAGCGATGGACCCACGTAAACCACATCTGAATAAACCTGAGGTAACTCCTACAGATGACCAGCCTTCCGCTGAGACAATCGAAGGTGTCACTAAGCCTTCCCACTACATGCTGTTTGACGACATTGAGGCTATCGAAGTGATTGCTCGTTCAATGACCGTTGAGCAGTTCAAGGGATACTGCTTCGGTa      <  1:129179/301‑1 (MQ=255)`  
                                                                                                                                                                                                                                                                                                         `ataatGAAGGTCGCCCAGATGATTTGAATGGCTGCGGTATTCTCTGCTCCAA‑TGTCCCTTGCCACCTCTGCCCCGCAAATAACGATCAAAAGATAACCTTAGGTGAAATCCGAGCGATGGACCCACGTAAACCACATCTGAATAAACCTGAGGTAACTCCTACAGATGACCAGCCTTCCGCTGAGACAATCGAAGGTGTCACTAAGCCTTCCCACTACATGCTGTTTGACGACATTGAGGCTATCGAAGTGATTGCTCGTTCAATGACCGTTGAGCAGTTCAAGGGATACTGCTTCGGTaa     >  1:202624/1‑301 (MQ=255)`  
                                                                                                                                                                                                                                                                                                         `aaaatGAAGGTCGCCCAGATGATTTGAATGGCTGCGGTATTCTCTGCTCCAA‑TGTCCCTTGCCACCTCTGCCCCGCAAATAACGATCAAAAGATAACCTTAGGTGAAATCCGAGCGATGGACCCACGTAAACCACATCTGAATAAACCTGAGGTAACTCCTACAGATGACCAGCCTTCCGCTGAGACAATCGAAGGTGTCACTAAGCCTTCCCACTACATGCTGTTTGACGACATTGAGGCTATCGAAGTGATTGCTCGTTCAATGACCGTTGAGCAGTTCAAGGGATACTGCTTCGGTaa     <  1:126382/299‑1 (MQ=255)`  
                                                                                                                                                                                                                                                                                                           `aatGAAGGTCGCCCAGATGATTTGAATGGCTGCGGTATTCTCTGCTCCAA‑TGTCCCTTGCCACCTCTGCCCCGCAAATAACGATCAAAAGATAACCTTAGGTGAAATCCGAGCGATGGACCCACGTAAACCACATCTGAATAAACCTGAGGTAACTCCTACAGATGACCAGCCTTCCGCTGAGACAATCGAAGGTGTCACTAAGCCTTCCCACTACATGCTGTTTGACGACATTGAGGCTATCGAAGTGATTGCTCGTTCAATGACCGTTGCGCAGTTCAAGGGATACTGCTTCGGTAACa   >  1:241953/1‑301 (MQ=255)`  
                                                                                                                                                                                                                                                                                                           `aatGAAGGTCGCCCAGATGATTTGAATGGCTGCGGTATTCTCTGCTCCAA‑TGTCCCTTGCAACCTCTGCCCCGCAAATAACGATCAAAAGATAACATTAGGTGAAATCCGAGCGATGGACCCACGTAAACAAAAACTGAATAAACCTGAGGTAACTCCTACAGATGACCAGCCTTCAGCTGAGACAAACGACGGTGTCACTAAGCCTTCCCACTTAATGCTGTTTGACGACATTGAGGCTATCGCAGTGATTGCTCGTTCAATGCCCGTTTAGCAGTTCAAGGGATCATGCTTCGGTAACa   >  1:182365/1‑301 (MQ=255)`  

                                                                                                                                                                                                                                                                                                            `|`                                                                                                                                                                                                                                                                                                               
`AGCGTCCACATCATTATCACCCGTGGTGACTTTGAGAAAGCAATAGACAAGAAACGTCCGGTTCTTAGTGTGGCAGTGACCCGCTTCCCGTTCGTCCGTCTGTTACTCAAACGAATCAAGGAGGTGTTCTGATGGGACTGTTAGATGGTGAAGCCTGGGAAAAAGAAAACCCGCCAGTACAAGCAACTGGGTGTATAGCTTGCTTAGAGAAAGATGACCGTTATCCACACACCTGTAACAAAGGAGCTAACGATATGACCGAACGTGAACAAGAGATGATCATTAAGTTGATAGACAATAATGAAGGTCGCCCAGATGATTTGAATGGCTGCGGTATTCTCTGCTCCAA‑TGTCCCTTGCCACCTCTGCCCCGCAAATAACGATCAAAAGATAACCTTAGGTGAAATCCGAGCGATGGACCCACGTAAACCACATCTGAATAAACCTGAGGTAACTCCTACAGATGACCAGCCTTCCGCTGAGACAATCGAAGGTGTCACTAAGCCTTCCCACTACATGCTGTTTGACGACATTGAGGCTATCGAAGTGATTGCTCGTTCAATGACCGTTGAGCAGTTCAAGGGATACTGCTTCGGTAACAT  >  NC_001604.1/8035‑8635`

  

Alignment Legend

Aligned base mismatch/match (shaded by quality score): `ATCG/ATCG` `< 3 ≤` `ATCG/ATCG` `< 10 ≤` `ATCG/ATCG` `< 21 ≤` `ATCG/ATCG` `< 34 ≤` `ATCG/ATCG` `< 38 ≤` `ATCG/ATCG`

Unaligned base: `atcg`    Masked matching base: `atcg`    Alignment gap: `‑`    Deleted base: `‑`

Reads not counted as support for junction

read\_name Not counted due to insufficient overlap past the breakpoint.

read\_name Not counted due to not crossing MOB target site duplication.
